# Antiviral Biologic Produced in DNA Vaccine/Goose Platform Protects Hamsters Against Hantavirus Pulmonary Syndrome When Administered Post-exposure

**DOI:** 10.1371/journal.pntd.0003803

**Published:** 2015-06-05

**Authors:** Nicole Haese, Rebecca L. Brocato, Thomas Henderson, Matthew L. Nilles, Steve A. Kwilas, Matthew D. Josleyn, Christopher D. Hammerbeck, James Schiltz, Michael Royals, John Ballantyne, Jay W. Hooper, David S. Bradley

**Affiliations:** 1 Department of Basic Sciences, University of North Dakota School of Medicine and Health Sciences (UND SMHS), Grand Forks, North Dakota, United States of America; 2 Virology Division, United States Army Medical Research Institute of Infectious Diseases (USAMRIID), Ft. Detrick, Maryland, United States of America; 3 Avianax, LLC, Grand Forks, North Dakota, United States of America; 4 Cedar Industries, Pierce, Colorado, United States of America; 5 Aldevron, Fargo, North Dakota, United States of America; Centers for Disease Control and Prevention, UNITED STATES

## Abstract

Andes virus (ANDV) and ANDV-like viruses are responsible for most hantavirus pulmonary syndrome (HPS) cases in South America. Recent studies in Chile indicate that passive transfer of convalescent human plasma shows promise as a possible treatment for HPS. Unfortunately, availability of convalescent plasma from survivors of this lethal disease is very limited. We are interested in exploring the concept of using DNA vaccine technology to produce antiviral biologics, including polyclonal neutralizing antibodies for use in humans. Geese produce IgY and an alternatively spliced form, IgYΔFc, that can be purified at high concentrations from egg yolks. IgY lacks the properties of mammalian Fc that make antibodies produced in horses, sheep, and rabbits reactogenic in humans. Geese were vaccinated with an ANDV DNA vaccine encoding the virus envelope glycoproteins. All geese developed high-titer neutralizing antibodies after the second vaccination, and maintained high-levels of neutralizing antibodies as measured by a pseudovirion neutralization assay (PsVNA) for over 1 year. A booster vaccination resulted in extraordinarily high levels of neutralizing antibodies (i.e., PsVNA_80_ titers >100,000). Analysis of IgY and IgYΔFc by epitope mapping show these antibodies to be highly reactive to specific amino acid sequences of ANDV envelope glycoproteins. We examined the protective efficacy of the goose-derived antibody in the hamster model of lethal HPS. α-ANDV immune sera, or IgY/IgYΔFc purified from eggs, were passively transferred to hamsters subcutaneously starting 5 days after an IM challenge with ANDV (25 LD_50_). Both immune sera, and egg-derived purified IgY/IgYΔFc, protected 8 of 8 and 7 of 8 hamsters, respectively. In contrast, all hamsters receiving IgY/IgYΔFc purified from normal geese (n=8), or no-treatment (n=8), developed lethal HPS. These findings demonstrate that the DNA vaccine/goose platform can be used to produce a candidate antiviral biological product capable of preventing a lethal disease when administered post-exposure.

## Author Summary

Our studies show the utility of combining DNA vaccination with the goose platform for the development of polyclonal avian antibodies for use as candidate medical countermeasures. We demonstrate that these antibodies have potent anti-viral neutralizing activity in cell culture and are efficacious in preventing hantavirus pulmonary syndrome in Syrian hamsters when administered as a post-exposure prophylactic. The polyclonal anti-Andes virus antibodies were not effective if administered late in the disease course indicating that the effective use of an avian polyclonal antibody-based approach to preventing hantavirus disease will require rapid diagnosis and treatment of persons presenting signs of hantavirus disease.

## Introduction

Andes virus (ANDV) is a New World hantavirus from the genus *Hantavirus* within the family *Bunyaviridae*, an etiological agent of hantavirus pulmonary syndrome (HPS). Hantaviruses are enveloped viruses with trisegmented single-stranded, negative-sense RNA genomes. The three genome segments S, M, and L encode for three structural proteins: the nucleocapsid (N) protein, two glycoproteins G_n_ and G_c_, and an RNA-dependent RNA-polymerase (RdRp), respectively [1]. ANDV was first reported and identified in southwestern Argentina in the mid-1990s [2,3], and since then outbreaks of HPS have occurred throughout South and Central America including Brazil, Chile, and Uruguay [4,5]. Most of these HPS cases are caused by ANDV, or ANDV-like viruses. Hantaviruses persist within rodents; whereas humans most likely become infected by inhalation or ingestion of virus containing urine or feces or by exposure to saliva through a bite from an infected rodent. ANDV is the only hantavirus known to be transmitted person-to-person [6,7]. Clinical HPS is characterized by a progression from flu-like symptoms and fever to non-cardiogenic pulmonary edema caused by vascular leakage. In fatal cases it is common for cardiogenic shock to develop [8]. The case fatality rate for HPS is 35–40% [4]. Despite the high mortality rate and the potential for person-to-person transmission, there are presently no approved vaccines, post-exposure prophylactics, or therapeutic treatments for HPS.

Studies emphasize the importance of the humoral immune response in hantavirus disease outcome support the use of antibodies as a potential treatment option for ANDV infection. In HPS cases, higher neutralizing antibody titers in patient’s serum have been shown to correlate with mild disease outcome [9]. Also, higher hantavirus specific IgG levels early in disease have been associated with survival [10]. In other hantavirus infections, hantavirus neutralizing activity has been related to antibodies directed to the surface glycoproteins, since monoclonal antibodies to G_n_ and G_c_ but not to N, have been shown to neutralize viral infection *in vitro* [11]. Specific to ANDV, a DNA vaccine expressing the M genome segment of the virus has been developed [12]. When either rhesus macaques or rabbits are vaccinated with this DNA vaccine, high-titer neutralizing antibodies are produced. Serum from these vaccinated animals, when passively transferred to ANDV-infected Syrian hamsters, protected the hamsters from lethal disease when given either before or after ANDV challenge [12,13]. It has also been shown that fresh frozen plasma (FFP) from convalescent HPS patients infected with ANDV was able to protect in the ANDV/Syrian hamster model when administered before or post-exposure [14]. In the post-exposure studies, it was necessary to administer the antibodies before high levels of serum viremia were detectable.

As an alternative to using human convalescent sera or mammalian antibodies as a source of neutralizing antibodies against ANDV, which are in short supply or have risks of reactogenicity in humans, antibodies against ANDV were purified from the eggs of ducks vaccinated with the ANDV DNA vaccine [14]. The α-ANDV IgY/IgYΔFc antibodies purified from the duck eggs were able to protect Syrian hamsters when administered after ANDV challenge [14]. An advantage to using IgY over human serum or other mammalian antibodies is the potential to prevent adverse reactions that the mammalian antibodies can have in the human body. Birds produce three different antibody isotypes: IgM, IgA, and IgY. IgY is the primary serum immunoglobulin of birds. It is the functional equivalent of mammalian IgG, but the two antibodies differ structurally. The advantage of IgY when used as a treatment include the inability to activate mammalian complement [15–20], interact with mammalian Fc receptors, or other receptors that are Fc binding, that could mediate an inflammatory reaction [21–26]. In addition to full length IgY, an alternatively spliced form of IgY, known as IgYΔFc, can be found in anseriformes birds e.g. geese and ducks, lacking the last two constant domains of the heavy chain [22,27]. In ducks, when immunized, the ratio of IgY to IgYΔFc shifts from predominantly IgY to predominantly IgYΔFc [28]. And indeed, a combination of IgY and IgYΔFc pass from mother to offspring via yolk-sac transmission [29]. Biologic and immunochemical studies have shown that IgYΔFc retains the ability to neutralize [28,30]. IgYΔFc from the eggs of vaccinated ducks or geese provide a unique source of neutralizing antibodies that is unobtainable from chicken eggs (express only full-length and membrane receptor forms of IgY) [28,31].

The transition from duck/egg to goose/egg was made based on preliminary studies showing increased frequency of high-titer responses in DNA-vaccinated geese versus ducks. Other advantages included a well-defined lineage of birds, 30+ generation breeding stock, expertise in breeding/hatching/goose husbandry, and the ability to collect more mg of antibody per yolk.

Here, we used geese vaccinated with the ANDV DNA vaccine to determine the post-exposure potential of goose-derived α-ANDV antibodies. The neutralizing capabilities of α-ANDV IgY/IgYΔFc purified from egg yolks of DNA vaccinated geese were determined after initial and long-term booster vaccinations. In addition, we identified epitopes within the ANDV glycoproteins recognized by IgY/IgYΔFc purified from egg yolks of DNA vaccinated geese. We show here, that neutralizing α-ANDV titers were maintained during the time period between initial and booster vaccinations, and that neutralizing titers were further increased after long-range booster vaccination. Finally, we demonstrate that polyclonal neutralizing antibodies produced using the DNA vaccine/goose platform administered post-exposure are capable of preventing disease in the ANDV/Syrian hamster model of lethal HPS.

## Results

### Vaccination of geese with ANDV DNA vaccine using a disposable syringe jet injection (DSJI) device elicited high-titer neutralizing antibody responses, particularly after long-range boost

**Fig 1:**
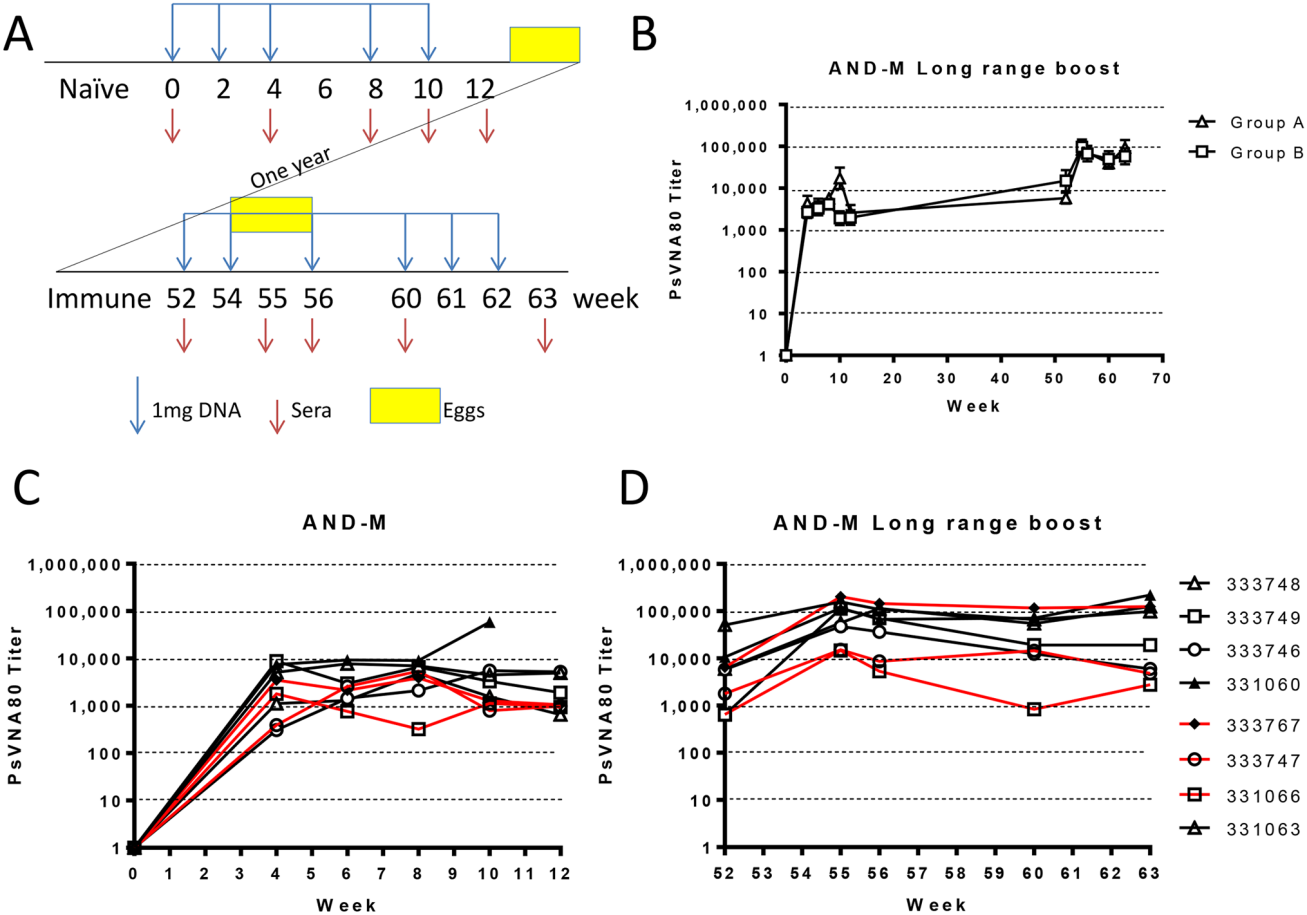
ANDV DNA vaccine is immunogenic in geese. **A**. Eight geese were vaccinated i.m. with 1mg ANDV DNA vaccine pWRG/AND-M(opt) or pWRG/AND-M(1.1) at 2 week intervals (blue arrows) using the PharmaJet V1. One year later the same, geese were booster vaccinated again with 1mg ANDV DNA pWRG/AND-M(opt) or pWRG/AND-M(opt2). Sera was collected from the vaccinated geese during the times indicated by red arrows. Egg collection is indicated by yellow ovals. **B.** Goose serum titers following initial vaccination series and **C.** long-range boost by PsVNA. Black lines indicate geese boosted with pWRG/AND-M(opt) and red lines indicate hamsters boosted with pWRG/AND-M(opt2). **D.** Mean of geese vaccinated initially with pWRG/AND-M and boosted with pWRG/AND-M(opt) (Group A) and pWRG/AND-M(opt2) (Group B).

Geese were vaccinated with an ANDV DNA vaccine, either pWRG/AND-M(opt) for Group A or pWRG/AND-M(1.1) for Group B, both containing the full-length M genome segment of ANDV strain Chile- 9717869. The difference between the plasmids used has been described previously [32]. The initial vaccination series included intramuscular vaccination at two-week intervals up to10 weeks. The first generation PharmaJet DSJI (blue) device was used for all vaccinations. One year later, the immune geese were boosted with the original or codon-optimized version of the AND-M vaccine, denoted either pWRG/AND-M(opt) or pWRG/AND-M(opt2) at week 52, 54, 56, 60, 61, and 62 (Fig 1A). Sera were collected from the vaccinated geese throughout the initial vaccination series and the long-range booster vaccination series. Sera neutralizing antibody titers were determined using the pseudovirion neutralization assay (PsVNA) [32]. Individual geese titers are shown for the initial vaccination period (Fig 1C) and the long-range boost (Fig 1D). The mean of Group A (receiving the pWRG/AND-M(opt) boost) and Group B (receiving the pWRG/AND-M(opt2) boost) indicate an order of magnitude increase in titer after the long-range boost. PsVNA neutralization 80% PsVNA80) titers as high as 100,000 were produced in the serum of vaccinated geese. Similarly, plaque reduction neutralization (PRNT) 50% titers of 20,480 were produced (S1 Fig). The PRNT assay is similar to the PsVNA (to obtain neutralizing antibody titers); however, ANDV is used rather than an ANDV pseudovirion. There was no significant difference between the AND-M DNA vaccines used during the initial vaccination series, or the long-range boost (Fig 1B).

### Purification of polyclonal antibodies from goose eggs results in a combination of both IgY and IgYΔFc

**Fig 2:**
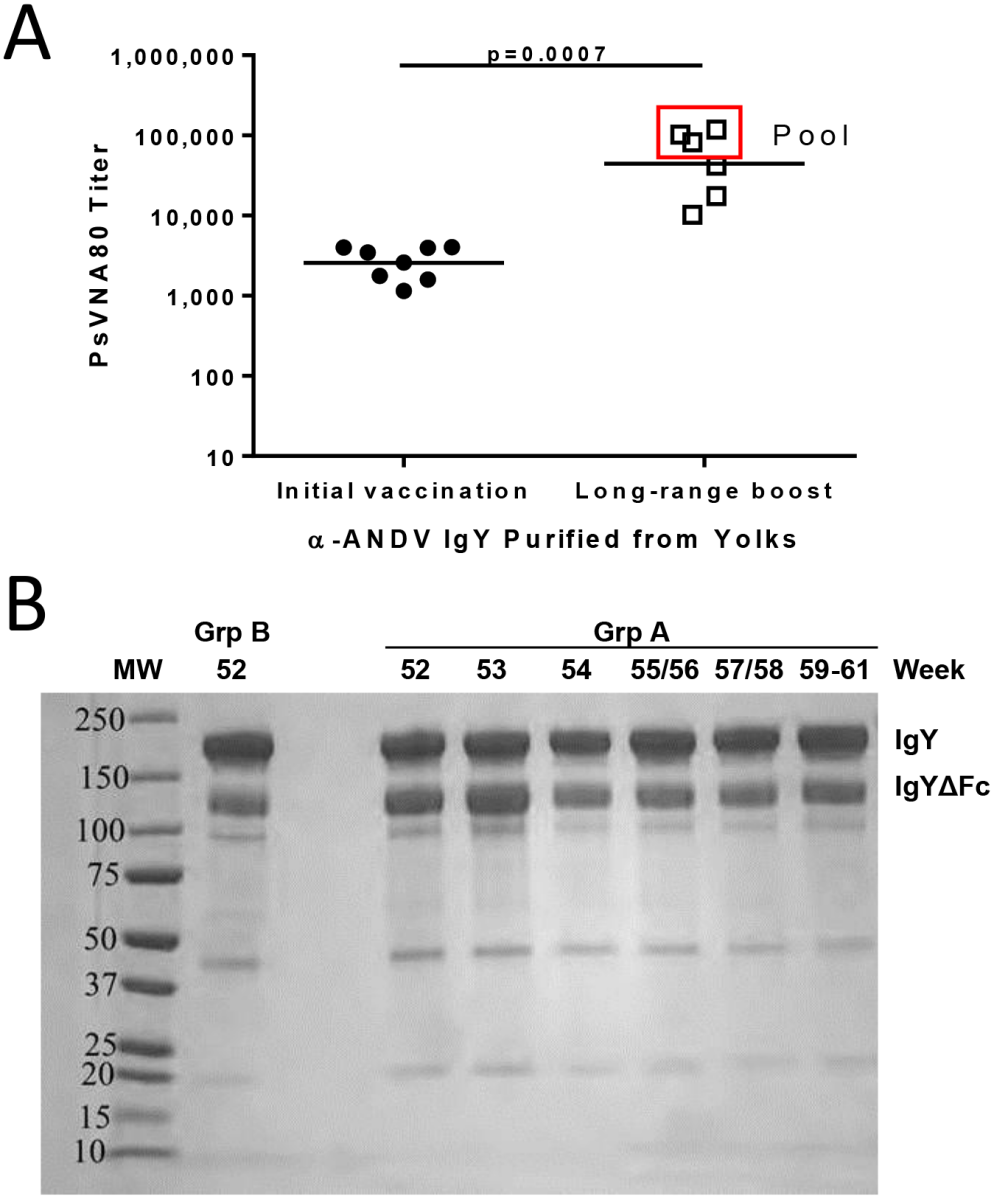
IgY/IgYΔFc from eggs collected from vaccinated geese has high-titer neutralizing activity. **A.** Total IgY was purified from eggs collected after the initial vaccination series and long-range boost and evaluated for α-ANDV neutralizing activity by PsVNA. The red box indicates samples that were pooled for future animal experiments. **B.** Purified IgY was visualized by Coomassie stain.

Goose eggs were collected after the initial vaccination and immediately after the long-range boost (following week 54 vaccination) (Fig 1A). Total IgY was purified from eggs yolks and evaluated for α-ANDV neutralizing activity by PsVNA. Similar to serum collected from the vaccinated geese, eggs collected after long-range boost showed an order of magnitude increase in neutralization titer (p = 0.0007) (Fig 2A). A pool was made containing purified IgY with the highest neutralization titer for animal experiments. The PRNT80 titer of the egg-derived IgY/ IgYΔFc was 2,560 and the PsVNA80 was 60,231, with a final protein concentration of 12.5 mg/ml. The yield of purified IgY is between 50–160 mg/yolk. Purified IgY from eggs (98% purity) collected at time points throughout the long-range boost were visualized by Coomassie staining (Fig 2B). Both IgY and IgYΔFc are detectable in eggs collected from Group A and Group B-vaccinated geese. Under nonreducing conditions, IgY is detected at approximately 180 kDa and IgYΔFc is detected at approximately 120 kDa (S2A Fig). Lower molecular weight bands were tested by Western blot using antibodies specific for IgY heavy and light chain subunits and found to correspond to the IgY light chain and heavy chains from IgY and IgYΔFc (S2B and S2C Fig). Total IgY isolated from eggs collected from geese from Group A show a shift in the ratio of IgY to IgYΔFc towards a preponderance of full-length IgY later in the long-range boost vaccination series.

### Goose egg IgY is specific to ANDV and recognizes unique epitopes when compared to IgG from vaccinated rabbits or IgG from human convalescent plasma

To determine the specificity of the IgY/IgYΔFc antibodies purified from the egg yolks of geese vaccinated with the ANDV M gene-based DNA vaccine, linear epitope mapping was completed using microarray slides. The M genome segment is the primary viral component of the ANDV DNA vaccine; two glycoproteins G_n_ and G_c_ are synthesized from the M segment and are present as oligomers on the outside surface of mature ANDV virions. To identify potential linear epitopes of ANDV glycoproteins G_n_ and G_c_ recognized by α-ANDV goose IgY and IgYΔFc, microarray slides were covalently linked with 13-mer peptides with a 10 amino acid overlap, and 3 amino acid offset, for a total of 376 peptides, spanning the entire sequence of the glycoproteins from ANDV strain Chile-9717869 (GenBank accession number AF291703). IgY, IgYΔFc, or IgY/IgYΔFc purified from the eggs of geese vaccinated at both initial and booster vaccination time points, as described in the Methods section, were incubated with microarray slides. Reactivity was compared to negative control features on the slide that did not contain any protein; the average of the negative controls was taken and used for comparison.

**Fig 3:**
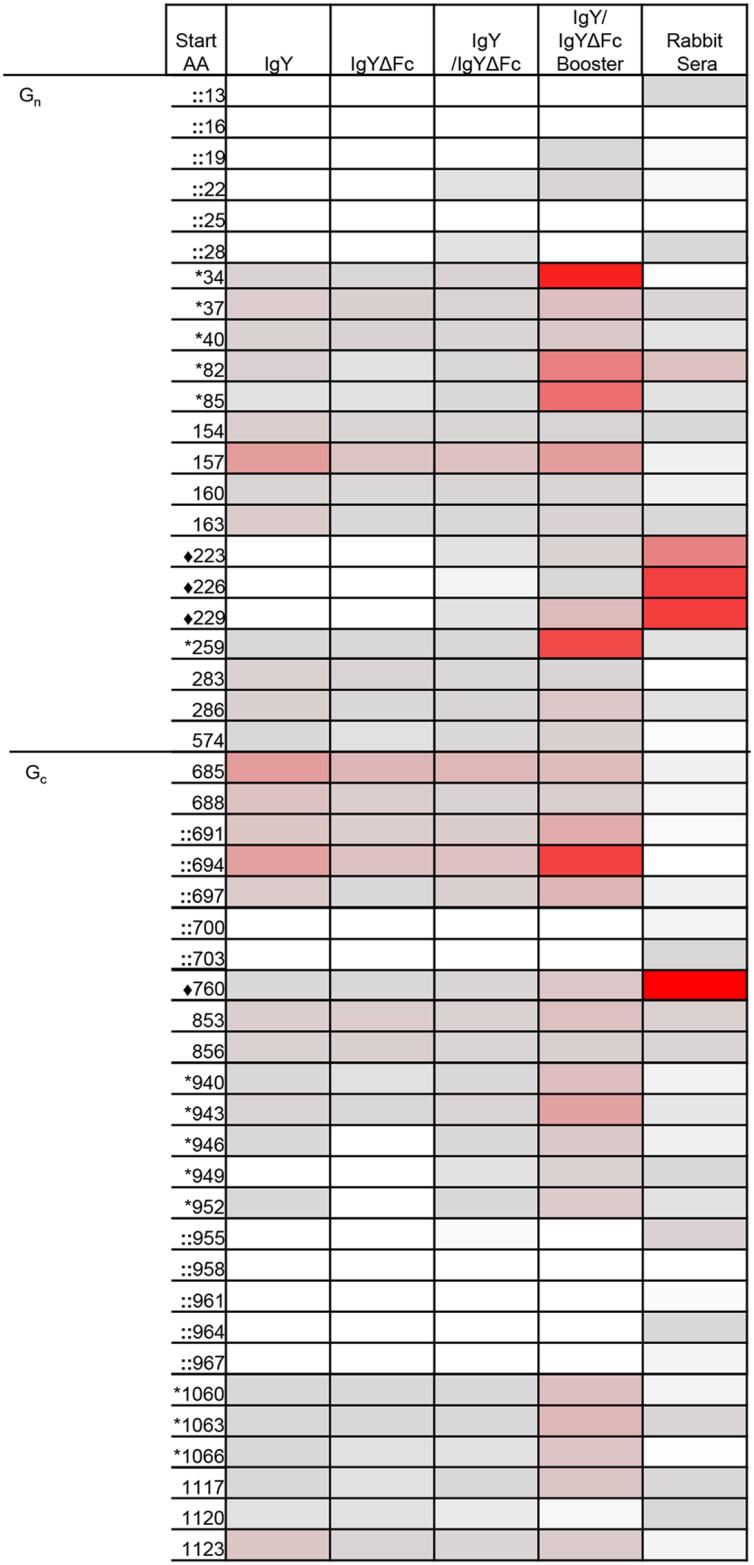
Identification of ANDV glycoprotein G_n_ and G_c_ epitopes. Microarray slides were incubated with IgY, IgYΔFc, or IgY/IgYΔFc isolated for egg yolks of vaccinated either after the initial or booster vaccination series, or serum from vaccinated rabbits (first column- amino acid peptide starts with, second column- IgY from egg yolks after initial vaccination series, third column- IgYΔFc from egg yolks after initial vaccination series, fourth column- IgY/IgYΔFc from egg yolks after initial vaccination series, fifth column- IgY/IgYΔFc from egg yolks after booster vaccination, sixth column- sera from vaccinated rabbit). Reactivity was measured based on a spectrum ranging from no activity in white, mild reactivity in gray, to strong reactivity in red. Epitopes regions marked with a (*) indicate epitopes of high interest after long-range booster vaccination. (::) indicate previously published amino acid regions with which human sera from HPS patients were strongly reactive [34]. (♦) indicate unique epitopes that rabbit IgG recognized strongly compared to IgY/IgYΔFc from eggs of vaccinated geese.

Eleven IgY/IgYΔFc reactive epitopes were identified across both glycoproteins G_n_ and G_c_ (Fig 3) outside of regions recognized by normal goose IgY/IgYΔFc. Six of the epitopes were specific for the G_n_ glycoprotein, peptides starting with aa 34–40, 82–85, 154–163, 259, 283–286, and 574 and the remaining five epitopes were within the G_c_ glycoprotein, reactive peptides started with aa 685–697, 853–856, 940–952, 1060–1066, and 1117–1123. When comparing the epitopes recognized by IgY/IgYΔFc, to the separated IgY or IgYΔFc, all goose antibody preparations recognized the same epitopes with similar levels of reactivity. There was no reactivity detected with any negative control slide features.

Because geese receiving the long-range booster vaccination showed increased IgY neutralizing antibody titers compared to the initial vaccination, we wanted to see if there was any change to the epitopes recognized by IgY/IgYΔFc from the geese receiving the long-range boost. Fig 3 shows that IgY/IgYΔFc from the egg yolks of the long-range boosted geese reacted with all of the same epitopes as the IgY/IgYΔFc from the initial vaccination and reactivity was either at the same level or increased. Areas of special interest because of increased activity after booster vaccination include areas starting with aa 34–40, 82–85, 259, 940–952, and 1060–1066 (Fig 3).

Sera from rabbits that were previously vaccinated with pWRG/AND-M [33] were also incubated with microarray slides to determine potential epitopes recognized by mammalian antibodies generated after vaccination. Most of the rabbit sera IgG antibodies recognized the same epitopes as IgY/IgYΔFc. However, in the majority of cases there was a noticeable difference in the level of binding of the rabbit IgG when compared to the IgY/IgYΔFc. There was increased binding to the peptide starting with aa 313 in G_n_. There was decreased binding to peptides starting with aa 685–697 (Fig 3). When comparing the rabbit IgG to the IgY/IgYΔFc from the eggs of booster vaccinated geese, there was one obvious region of decreased reactivity in G_n_ corresponding to the peptide starting with aa 34 (Fig 3). There were two unique epitopes recognized strongly by the rabbit IgG compared to IgY/IgYΔFc from the eggs of vaccinated geese comprised of peptides starting with aa 223–229 in G_n_ along with peptide 760 in G_c_ (Fig 3).

Important protective epitopes are most likely the five areas with increased IgY/IgYΔFc binding after booster vaccination. These five regions were also recognized by the rabbit sera, but to a lesser reactivity level than IgY/IgYΔFc from booster vaccinated geese and all IgY treatments (IgY, IgYΔFc, and IgY/IgYΔFc) for some regions. All five regions with increased reactivity are outside of the regions identified as reactive by human sera from ANDV patients, and sera from naturally infected rodents [34]. Epitopes in the study by *Tischler et al*. characterized as having strong reactivity for serum from humans ANDV HPS patients across both G_n_ and G_c_ were aa 14–26, 691–703, and 955–967. The strongly reactive epitopes for serum from naturally infected rodents were aa 599–611 within G_n_ and aa 691–703, 918–30, and 955–967 within G_c_ [34]. In addition, the highly reactive IgY epitopes do not overlap with regions recognized by monoclonal antibodies that neutralize other hantaviruses [35–37]. Taken together these results show that IgY isolated from eggs yolks of vaccinated geese was ANDV specific and recognized unique epitopes compared to human and rabbit serum.

### Bioavailability of α-ANDV IgY/IgYΔFc indicates repeating dosing for passive transfer experiments

**Fig 4:**
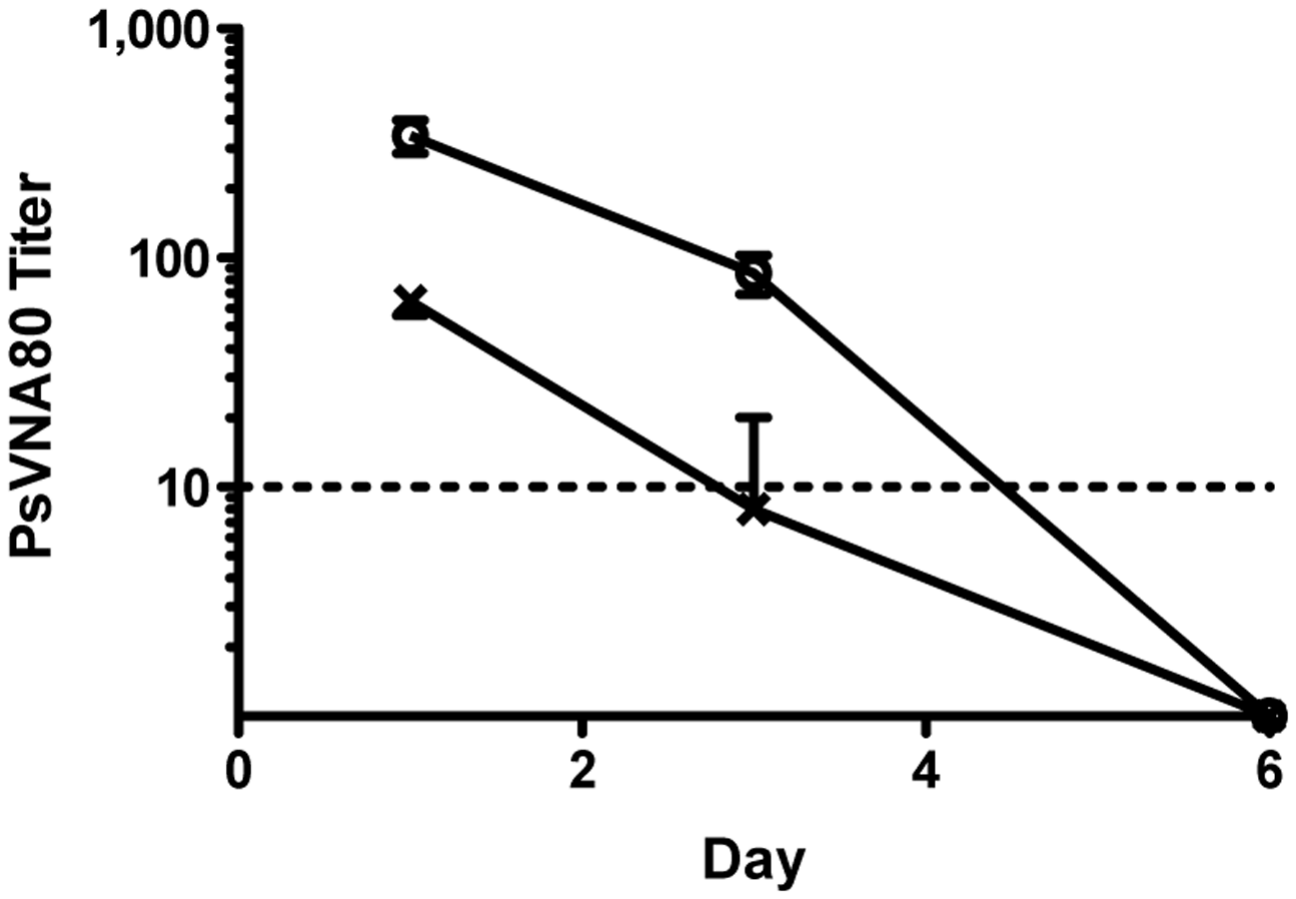
Bioavailability of α-ANDV IgY/IgYΔFc in Syrian hamsters. Groups of three hamsters were injected with 64,000 NAU/kg (open circles) or 12,000 NAU/kg (crosses) α-ANDV IgY/IgYΔFc by the s.c. route. Sera collected on days 1, 3, and 6 were evaluated for ANDV neutralizing activity by PsVNA. Each point represents the mean PsVNA80 titer ±SE. The limit of detection for this assay is represented by a dashed line.

Having produced potent α-ANDV neutralizing antibodies using the goose platform, we were interested in testing the efficacy of this antiviral biologic in an animal model of hantavirus disease. Before performing a protection study in the ANDV/Syrian hamster model of lethal HPS, a bioavailability experiment was performed testing the goose-generated IgY/IgYΔFc in hamsters (Fig 4). Groups of three hamsters were injected by the subcutaneous route with 64,000 NAU/kg (neutralizing antibody units/kilogram) or 12,000 NAU/kg α-ANDV IgY/IgYΔFc. Serum samples were collected on days 1, 3, 6, 9, 15, and 21 after antibody injection, and titers were assessed by PsVNA. α-ANDV neutralizing antibodies were detected on days 1 and 3 for the group of hamsters receiving 64,000 NAU/kg α-ANDV IgY/IgYΔFc, then dropped below the level of detection for the assay. On day 3, only a single hamster from the group receiving 12,000 NAU/kg α-ANDV IgY/IgYΔFc had a positive neutralizing antibody titer, which was reduced compared to the high dosage group. These findings were used to determine the dose and schedule of antibody injections in protection experiments described below.

### 20,000 NAU/kg α-ANDV goose sera and goose IgY/IgYΔFc administered as a post-exposure prophylactic protects hamsters from lethal ANDV challenge

**Fig 5:**
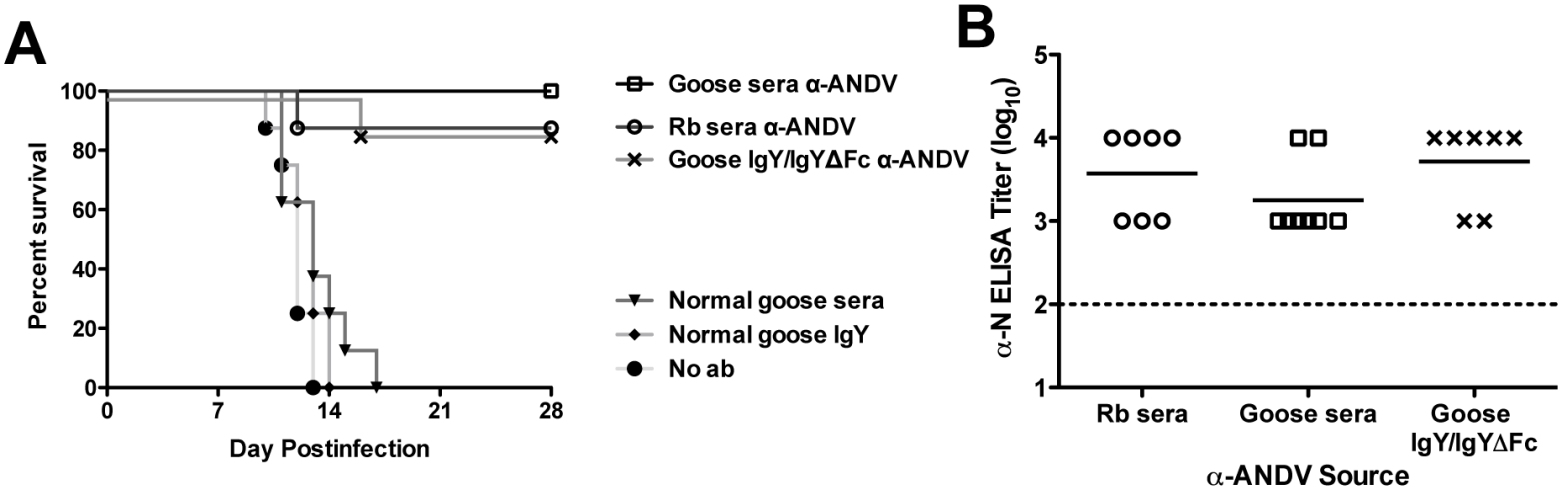
α-ANDV IgY/IgYΔFc administered prior to the onset of viremia protects hamsters from lethal HPS. **A.** Survival curve of hamsters that were challenged with 200 PFU i.m. of ANDV on day 0 and passively transferred with 20,000 NAU/kg α-ANDV goose sera, 20,000 NAU/kg α-ANDV goose IgY/IgYΔFc, 20,000 NAU/kg α-ANDV rabbit sera, normal goose sera, or normal goose IgY on days 5 and 8 post-infection (grey arrows). n = 8 for all groups. **B.** α-N ELISA endpoint titers (log_**10**_) were conducted with sera from surviving hamsters challenged with ANDV in A. GMT for each group are shown. The limit of detection, a titer of 100, is shown as a dotted line.

We next determined the protective efficacy of goose-generated α-ANDV IgY/IgYΔFc in the hamster model of lethal HPS. On day 0, all hamsters were challenged with 200 PFU of ANDV by the i.m. route (Fig 5A). On day 5, groups of 8 hamsters were administered 12,000 NAU/kg α-ANDV rabbit sera as a positive control, 20,000 NAU/kg α-ANDV goose sera, 20,000 NAU/kg α-ANDV IgY/IgYΔFc, an equivalent dose of normal goose sera, or an equivalent protein concentration of normal goose IgY/IgYΔFc as negative controls. All antibody injections were by the s.c. route. On day 8, these same groups were administered a second treatment of 20,000 NAU/kg α-ANDV goose sera, 20,000 NAU/kg α-ANDV IgY/IgYΔFc, an equivalent dose of normal goose sera, or an equivalent dose of normal goose IgY/IgYΔFc by the s.c. route. All hamsters receiving normal goose sera, normal goose IgY/IgYΔFc, or no antibody treatment succumbed to HPS between days 10 and 17 postinfection displaying clinical signs of HPS (i.e. staggered gait, tachypnea). Of the α-ANDV treatment groups, 7/8 hamsters receiving positive control rabbit sera survived to day 28 (p<0.0001), 8/8 hamsters receiving α-ANDV goose sera survived to day 28 (p<0.0001), and 7/8 hamsters receiving α-ANDV goose-egg derived purified IgY/IgYΔFc survived to day 28 (p = 0.0003). None of the surviving hamsters displayed clinical signs of HPS. Sera from all surviving hamsters was collected on day 28 and subjected to an N-ELISA (Fig 5B). Results from the ELISA indicated that all hamsters were productively infected with ANDV, yet did not succumb to lethal HPS.

### 40,000 NAU/kg α-ANDV goose IgY/IgYΔFc administered after the onset of viremia does not protect hamsters from lethal ANDV challenge

**Fig 6:**
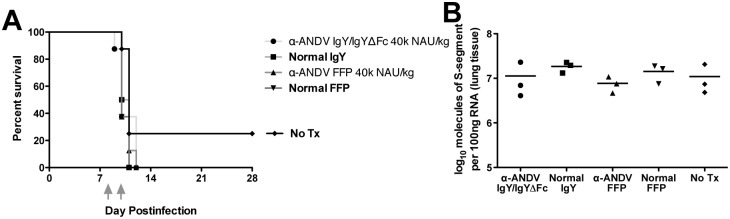
α-ANDV IgY/IgYΔFc administered after the onset of viremia does not protect hamsters from lethal HPS. **A.** Survival curve of hamsters that were challenged with 200 PFU i.m. of ANDV on day 0 and passively transferred with 40,000 NAU/kg α-ANDV IgY/IgYΔFc, 40,000 NAU/kg α-ANDV human FFP, normal IgY, or normal FFP on days 8 and 10 post-infection (grey arrows). n = 8 for all groups. **B.** Serum and lung isolated from ANDV-infected hamsters on day 10 were evaluated for viral genome by RT-PCR. Symbols represent lung tissue titers from different animals. The mean for each group is also shown (line).

Having evaluated α-ANDV IgY/IgYΔFc administered as a post-exposure prophylactic (prior to the onset of viremia in the ANDV hamster model), we next determined the efficacy of α-ANDV IgY/IgYΔFc when administered as a potential therapeutic (after the onset of viremia). For a 200 PFU i.m. ANDV challenge, the mean day-to-death is 11 days post-infection [14,33]. Therefore, treatment beginning on day 8 post-infection would be 2 days after the onset of viremia (day 6) and 3 days before the mean day-to-death. In this experiment, the dosage of α-ANDV neutralizing antibodies was doubled from 20,000 to 40,000 NAU/kg. In addition to α-ANDV IgY/IgYΔFc, α-ANDV FFP from a Chilean HPS survivor [38] was evaluated for its efficacy as a therapeutic. On day 0, all hamsters were challenged with 200 PFU of ANDV by the i.m. route (Fig 6A). On days 8 and 10, groups of hamsters were passively transferred with 40,000 NAU/kg α-ANDV IgY/IgYΔFc, 40,000 NAU/kg α-ANDV FFP, or equivalent protein concentration of normal IgY or equivalent dose normal FFP. A group of 8 hamsters were untreated to serve as an infection control. All hamsters from the α-ANDV IgY/IgYΔFc, α-ANDV FFP, normal IgY, and normal FFP succumbed to lethal disease between days 9–12 post-infection. Two hamsters from the ANDV infection only control group survived to day 28 (end of study). Sera from these hamsters were subjected to an N-ELISA demonstrating a productive infection (S3 Fig).

RNA isolated from the lungs of a subset of ANDV-infected hamsters on day 10 was evaluated for ANDV viral genome by RT-PCR (Fig 6B). These results show no statistically significant difference between any of the treatment groups and control groups. These findings indicate that a late treatment with a dose of α-ANDV antibodies, regardless of goose or human source, is insufficient to lower the amount of virus accumulating in the lung, and is unable to reverse the disease course in the hamster model.

## Discussion

ANDV has been associated with a majority of HPS cases [3] and continues to be the only hantavirus capable of human-to-human transmission. In spite of the continuing number of HPS cases and the staggering mortality rate of 35–40% there are no available treatments or preventative vaccines. The potential for the use of passive treatments to protect against HPS has already been established by previous studies utilizing immune serum from infected patients and isolated antibodies from vaccinated animals in the ANDV/hamster model [12–14,39]. In addition, evaluation of HPS patients has highlighted the importance of neutralizing antibody production in recovery from infection [9,10]. An obvious source of antibodies for passive treatment would be immune plasma from convalescent patients, but immune plasma is in short supply and can pose problems of reactogenicity when given to other patients if not blood group-typed appropriately. Protective monoclonal antibodies are another option for treatment and have been used with other viral infections [40], but thus far, ANDV neutralizing monoclonal antibodies have not been described. Polyclonal antibodies have been generated against toxins and venoms in vaccinated sheep and horses, but this method has yet to be successful in generating any antiviral treatments. Avian antibodies are yet another logical alternative source of passive therapeutics with the potential to overcome the shortcomings of current therapeutics antibodies; be it source shortages, reactivity to Fc portions of mammalian antibodies, or lack of reactivity with neutralizing epitopes. IgY is the primary serum antibody of birds and is transferred to the egg yolk via receptors on the surface of the yolk membrane that is specific for IgY translocation, causing the yolk to have high IgY concentrations [27,41–44]. IgY can then be purified from egg yolks and in large quantities for use as therapeutics. Current and previous research using polyclonal avian IgY has already established a baseline for its therapeutic potential against infectious agents e.g. *Pseudomonas aeruginosa* [22,45–47] and *Candida albicans* [48]. IgY antibodies have also been developed against different venoms and antitoxins [49–55]. Most important for this research, DNA vaccinated birds have also been used to produce virus-specific IgY [14,56,57].

DNA vaccination of ducks with pWRG/AND-M had already been shown to result in the production of ANDV neutralizing antibodies [14]. Here, geese were vaccinated and initial total IgY PsVNA80 titers from eggs were 2,567 (geometric mean titer, GMT from Fig 2). Long-range booster vaccination resulted in an increase in total egg IgY neutralizing antibody PsVNA80, titer GMT = 62,195. The increase in titers correlated with an increase in ANDV-specific neutralization PsVNA80 titers for IgY/IgYΔFc in sera. PsVNA80 titers for sera increased from >1,000 just 4 weeks into the initial vaccination series to >10,000 3 weeks into booster vaccination **(** Fig 1). We demonstrated that neutralizing antibody titers were maintained during the year between initial vaccination and booster vaccination. While unexpected, these results point out the advantage of long-range boost leading to an order of magnitude enhanced antibody response.

It is interesting to note the shift in IgY to IgYΔFc ratio to predominantly full-length IgY during the long-range boost vaccination series (Fig 2B). In an early publication detailing duck immunoglobulins, the author describes a shift from predominantly IgY to IgYΔFc in serum from hyper-vaccinated ducks [28]. We speculate that this difference could potentially be attributed to the use of a protein vaccine as opposed to a DNA vaccine. However, this difference could also be attributed to the species used. Following DNA vaccination, total IgY isolated from duck eggs contained 75% IgYΔFc [14]. Regardless, the shift towards IgY or IgYΔFc does not appear to have a differential effect in efficacy studies. Future experiments isolating α-ANDV full-length IgY from α-ANDV IgYΔFc will determine the contribution of Fc in protection studies.

Epitope mapping using IgY purified from egg yolks of vaccinated geese identified several regions of reactivity on the ANDV surface glycoproteins G_n_ and G_c_. It is likely that many epitopes recognized by ANDV neutralizing antibodies are conformational; however, it is possible that there are linear neutralizing epitopes as well. In total, following the initial vaccination there were 11 epitopes recognized. After the long-range booster vaccination there were five epitopes with increased reactivity. The increase in neutralizing capabilities of both sera from DNA vaccinated geese along with IgY/IgYΔFc isolated from egg yolks of vaccinated geese supports the protective potential of these five epitopes making these epitopes of high interest. Four of the five highly reactive regions in glycoproteins G_n_ and G_c_ did not overlap with regions previously identified as reactive by serum from human ANDV HPS patients or serum from naturally infected rodents [34]. The only overlap with human and rodent epitopes was at the epitope made up of aa 685–697. This region overlapped the strongly reactive rodent and human serum epitope made up of amino acids 691–703 [58]. This is a potentially immunodominant domain since it is being recognized by antibodies from multiple species.

Looking at the predicted structure of the ANDV glycoproteins as part of a mature virion, all five epitopes with increased reactivity after long-range boost are within regions potentially accessible from the surface of the virion. AA 34–40, 82–85, and 259 are part of the predicted G_n_ ectodomain prior to WAASA the cleavage site, found at aa 647–651, where the glycoprotein precursor is cleaved into the two glycoproteins G_n_ and G_c_ [58]. Peptides starting with aa 34–40 are near the n terminus of G_n_. The secondary structure of this region is predicted to be a mixture of α-helices and β strands. For the G_c_ glycoprotein highly reactive regions aa 940–952 and 1060–1066 are within the predicted G_c_ ectodomain of the protein with the secondary structure primarily being made of β-sheets and random coils. In addition all five of these regions are outside of predicted hydrophobic regions, or glycosylation sites of the glycoproteins [58]. These epitopes of high interest recognized by the IgY/IgYΔFc from the egg yolks of geese vaccinated with at long-range booster represent novel potentially neutralizing epitopes on the ANDV glycoproteins.

We have shown previously that neutralizing antibodies administered on day 5 following a 200 PFU ANDV i.m. challenge can protect hamsters from lethal HPS [14]. In that study, 75% of hamsters receiving 12,000 NAU/kg α-ANDV duck IgY/IgYΔFc survived ANDV infection. In order to bolster the survival percentage, 20,000 NAU/kg α-ANDV goose IgY/IgYΔFc was administered to hamsters on days 5 and 8 post-infection (based on bioavailability results). This resulted in 88% survival (7/8 hamsters). In a clinical setting, it is likely that increasing the neutralizing antibody per dose, and increased frequency of dosing, would increase the efficacy of any antibody-based treatment for HPS.

In order to evaluate the use of IgY/IgYΔFc as a therapeutic, an experiment was conducted to determine the efficacy of administering a high concentration of α-ANDV neutralizing antibody after the onset of viremia, which in the 200 PFU i.m. challenge starts on day 6 [59]. By doubling the concentration of antibodies delivered (from 20,000 NAU/kg to 40,000 NAU/kg) and starting treatment on day 8 post-infection, we were unable to protect ANDV-infected hamsters from lethal HPS. It is unknown if a higher concentration of neutralizing antibody alone, changing the route of administration, and/or a treatment starting on day 7, which would still be post-viremia, would be sufficient to achieve a survival outcome. Ribavirin, favipiravir (T-705), and neutralizing antibodies have all been proven efficacious prior to the onset of viremia [12,14,60,61]. To date, there is no treatment option for HPS that has demonstrated efficacy after the onset of viremia. This highlights the need for a treatment option that expands the current therapeutic window.

A compassionate open trial is underway in Chile using α-ANDV human FFP to treat ANDV infections. ABO-compatible immune plasma at a dosage of 5,000 NAU administered to confirmed hantavirus cases resulted in a reduction in the rate of lethality from 32% to 14% [62]. This is a borderline statistically significant reduction in lethality; however, it is suggestive of the potential clinical benefit of this type of therapy. We speculate that the timing of α-ANDV plasma therapy plays a critical role in the ability of the antibodies to neutralize virus and prevent morbidity and mortality. A greatly increased concentration of neutralizing antibodies (40,000 NAU/kg) was insufficient to protect hamsters from lethal HPS if administered 3 days prior to the mean day-to-death, further supporting the argument for early intervention in suspected HPS cases.

In this study we used the DNA vaccine/goose platform to produce a biologic consisting of purified IgY antibodies targeting both ANDV G_n_ and G_C_ envelope glycoproteins. This candidate product has potent anti-viral neutralizing activity and is effective at preventing disease when administered as a post-exposure prophylactic in the Syrian hamster model. Translating an egg-derived polyclonal antibody product to the clinic is a daunting challenge; however, the ongoing phase III clinical trial test the efficacy of avian polyclonal IgY antibodies against *Pseudomonas aeruginosa* for the treatment of cystic fibrosis under the auspices of the European Medicines Agency indicates that novel avian antibody-based approaches to the development of medicines to prevent and treat infectious disease has merit.

## Materials and Methods

### Virus and cells

A twice plaque-purified ANDV strain Chile-9717869 passaged in Vero E6 cells (Vero C1008, ATCC CRL 1586) was described previously [63]. Cells were maintained in Eagle’s minimum essential medium with Earle’s salts (EMEM) supplemented with 10% fetal bovine serum, 10nM HEPES (pH 7.4), 200 U/ml penicillin, 200 μg/ml streptomycin, 1X nonessential amino acids, 1.5 μg/ml amphotericin B, and 50 μg/ml gentamicin sulfate (cEMEM) at 37°C in a 5% CO_2_ incubator.

### DNA vaccines

pWRG/AND-M (1.1) has been described previously [13]. Details of codon-optimized DNA vaccines pWRG/AND-M(opt) and pWRG/AND-M(opt2) are contained in a separate manuscript [32]. Briefly, pWRG/AND-M(1.1) was generated using viral RNA from ANDV-infected Vero E6 cells. pWRG/AND-M(opt) is identical to pWRG/AND-M(1.1) with the ORF codon-optimized for *Homo sapiens* but missing a stop codon, resulting in the addition of 24 amino acids to the Gc C-terminus. pWRG/AND-M(opt2) is the corrected DNA vaccine.

### Geese and PharmaJet vaccination

Two groups containing four geese each (*Anser domesticus*, 25 months old) were immunized with the indicated AND-M DNA vaccine intramuscularly using the v1.0 PharmaJet injector. Five 1mg DNA inoculations were delivered to the breast at indicated times. The same delivery device and site was used for the long-range boost immunized at approximately 1 year after the initial immunization to match the start of the next goose laying season.

### Goose IgY/IgYΔFc purification from eggs

Yolks were isolated, rinsed with water, and punctured to drain the contents. These contents were diluted 1:10 with cold, deionized water, stirred, and acidified to pH 5.0. The diluted yolk was centrifuged at 10,000 x g for 30 min and supernatant was filtered. An equal volume of 100% saturated ammonium sulfate was added to the filtered supernatant to give a 50% saturation, stirred for 30 min, and centrifuged at 10,000 x g for 15 min. The pellet was suspended in 50 mM TrisCl pH 8.0. Further purification was achieved via hydrophobic charge induction chromatography on 4-Mercapto-Ethyl-Pyridine-lined (MEP) HyperCel sorbent (Pall Biosciences) followed by concentration using a Tangential flow filtration and diafilitration with 1x PBS buffer. This method results in a combination of full-length IgY and truncated IgYΔFc.

### Pseudovirion neutralization assay (PsVNA)

The PsVNA was run as previously described [32]. Briefly, a 1:10 dilution of heat-inactivated sera was made followed by five-fold serial dilutions that were mixed with equal volume of cEMEM containing 4,000 FFU ANDV PsV with 10% guinea pig complement. The mixture was incubated overnight at 4°C. Following this incubation, 50μl was inoculated onto Vero cell monolayers in a clear bottom black-walled 96-well plate (Corning) in triplicate. Plates were incubated at 37°C for 18–24 hrs. The media was discarded, and cells were lysed according to the luciferase kit protocol (Promega #E2820). A Tecan M200 Pro was used to acquire raw luciferase data. The values were graphed using GraphPad Prism software (version 6) to calculate the 80% neutralization and then interpolate to obtain PsVNA80 titers.

### SDS-PAGE

Duplicate IgY/IgYΔFc samples were separated by 4–15% gradient SDS-PAGE run under nonreducing conditions. The gel was incubated in Bio-Safe Coomassie G-250 stain (Bio-Rad Laboratories) for 30 minutes and subsequently destained (deionized water) over 2.0 hrs before visualized. Signals were captured using AlphaView software and AlphaImager HP Imaging System (Alpha Innotech).

### Epitope determination

Linear IgY epitopes were identified using JPT PepStar microarrays. The entire glycoprotein precursor sequence of the ANDV strain Chile-9717869 was synthesized into 13 amino acid peptides. The 376 resulting 13-mer peptides were covalently attached to a microarray slide with a 10 amino acid overlap. Peptides were analyzed for their reactivity with IgY antibodies isolated from geese eggs, following protocols recommended by JPT. Briefly, slides were incubated with 30μg/mL primary antibody at 4°C overnight in a moist environment. The slide was washed and incubated with a fluorescently labeled secondary antibody, goat anti-chicken IgY conjugated to Cy5 (1ug/mL) (Abcam, for 1 hour at 30°C. After washing and drying the slide, bound antibodies were detected using a microarray reader (Genepix 4000). Fluorescence was measured at a 10um pixel size and mean values with the background corrected were calculated and used for analysis. The reactivity was classified based on a spectrum ranging from no activity in white, mild reactivity in gray, to strong reactivity in red.

### Intramuscular injection of hamsters with virus

Female Syrian hamsters aged 6–8 weeks (Harlan) were anesthetized by inhalation of vaporized isoflurane using an IMPAC 6 veterinary anesthesia machine. Once anesthetized, hamsters were injected with 200 PFU of virus diluted in PBS. Intramuscular (i.m.) (caudal thigh) injections consisted of 0.2ml delivered with a 1ml syringe with a 25-gauge, 5/8in needle.

### Passive transfer of antibody to hamsters by subcutaneous injection

Hamsters were anesthetized by inhalation of vaporized isoflurane using an IMPAC 6 veterinary anesthesia machine. Once anesthetized, hamsters were injection with antibody diluted in PBS. Subcutaneous injections consisted of 1–2ml delivered with a 3ml syringe with a 23-gauge, 1in needle. Positive control rabbit sera was collected from rabbits vaccinated with pWRG/AND-M four times by muscle electroporation [33].

### N-specific ELISA

The enzyme-linked immunosorbent assay (ELISA) used to detect N-specific antibodies (N-ELISA) was described previously [64,65]. The endpoint titer was determined as the highest dilution that had an optical density (OD) greater than the mean OD for serum samples from negative-control wells plus 3 standard deviations. The PUUV N antigen was used to detect ANDV N-specific antibodies as previously reported [63].

### Isolation of RNA and real-time PCR

Approximately 250 mg of lung tissue was homogenized in 1.0 ml TRIzol reagent using gentleMACS M tubes and a gentleMACS dissociator on the RNA setting. RNA was extracted from TRIzol samples as recommended by the manufacturer. The concentration of the extracted RNA was determined using a NanoDrop 8000 instrument and standardized to a final concentration of 100 ng/ul. Real-time PCR was conducted on a BioRad CFX thermal cycler using an Invitrogen Power SYBR Green RNA-to-Ct one-step kit according to the manufacturer’s protocols. Primer sequences are ANDV S 41F 5’-GAA TGA GCA CCC TCC AAG AAT TG-3’ and ANDV S 107R 5’-CGA GCA GTC ACG AGC TGT TG-3’ [66]. Cycling conditions were 30 min at 48°C, 10 min at 95°C, followed by 35 cycles of 15 sec at 95°C and 1 min at 60°C. Data acquisition occurs following the annealing step.

### Statistical analysis

Comparison of egg IgY titers was done using Student’s t-test (two-tailed). *P* values of less than 0.05 were considered significant. Survival analyses were compared using Kaplan-Meier survival analysis with log rank tests and *P*-values adjusted by simulation or by Dunnett’s test to account for multiple comparisons. Analyses were conducted using GraphPad Prism (version 6).

### Ethics statement

The goose work was approved by the University of North Dakota Institutional Animal Care and Use Committee: Protocol Number 1403–1. The hamster work was approved by the USAMRIID Institutional Animal Care and Use Committee.

Research was conducted under an IACUC approved protocol in compliance with the Animal Welfare Act, PHS Policy, and other Federal statutes and regulations relating to animals and experiments involving animals. The USAMRIID is accredited by the Association for Assessment and Accreditation of Laboratory Animal Care, International and adheres to principles stated in the Guide for the Care and Use of Laboratory Animals, National Research Council, 2011. Opinions, interpretations, conclusions, and recommendations are ours and not necessarily endorsed by the U.S. Army or the Department of Defense.

## Supporting Information

S1 Fig Goose serum neutralizing antibody responses measured by plaque reduction neutralization test (PRNT). Goose serum samples collected on weeks 55 and 61 following the long-range boost were analyzed by PRNT for α-ANDV neutralizing activity as described previously [65].
(TIF)

S2 Fig Both IgY and IgYΔFc can be visualized by Coomassie stained SDS-PAGE and Western blot. Comparison of +DTT (reduced) and–DTT (non-reduced) IgY isolated from goose sera and egg (50mM DTT) visualized by A) Coomassie stained SDS-PAGE, B) IgY heavy chain Western blot, or C) IgY light chain Western blot. Note IgY concentration listed on Coomassie stained gel in A) was used for Western blots in B) and C). SDS-PAGE gel was transferred onto Immobilon-FL (Millipore), blocked with 1% BSA in TTBS, then incubated with 1:2000 dilution of either B) rabbit α-IgY heavy chain IgG or C) rabbit α-IgY light chain IgG. This was followed by a goat α-rabbit IgG linked to a biotin and a streptavidin linked to Q-dot 625 (Invitrogen). Signals were captured using AlphaView software and AlphaImager HP Imaging System (Alpha Innotech).
(TIF)

S3 Fig Surviving hamsters from ANDV infection only group were positive by N-ELISA, indicating a productive ANDV infection. Serum from the two hamsters surviving to day 28 following ANDV challenge were analyzed by N-ELISA (see Materials and Methods). Each symbol represents and individual animal. The limit of detection, a titer of 100, is shown as a dotted line.
(TIF)
