# Supplementary figures and images for: Antiviral Biologic Produced in DNA Vaccine/Goose Platform Protects Hamsters Against Hantavirus Pulmonary Syndrome When Administered Post-exposure
Source: PLoS Negl Trop Dis. 2015 Jun 5;9(6):e0003803. doi: 10.1371/journal.pntd.0003803 (PMC4457835; doi:10.1371/journal.pntd.0003803)

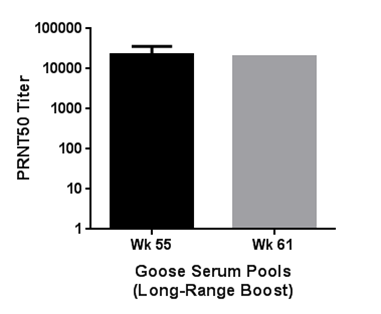

Supplement: S1 Fig — Goose serum samples collected on weeks 55 and 61 following the long-range boost were analyzed by PRNT for α-ANDV neutralizing activity as described previously [65]. (TIF) [file pntd.0003803.s001.tif]

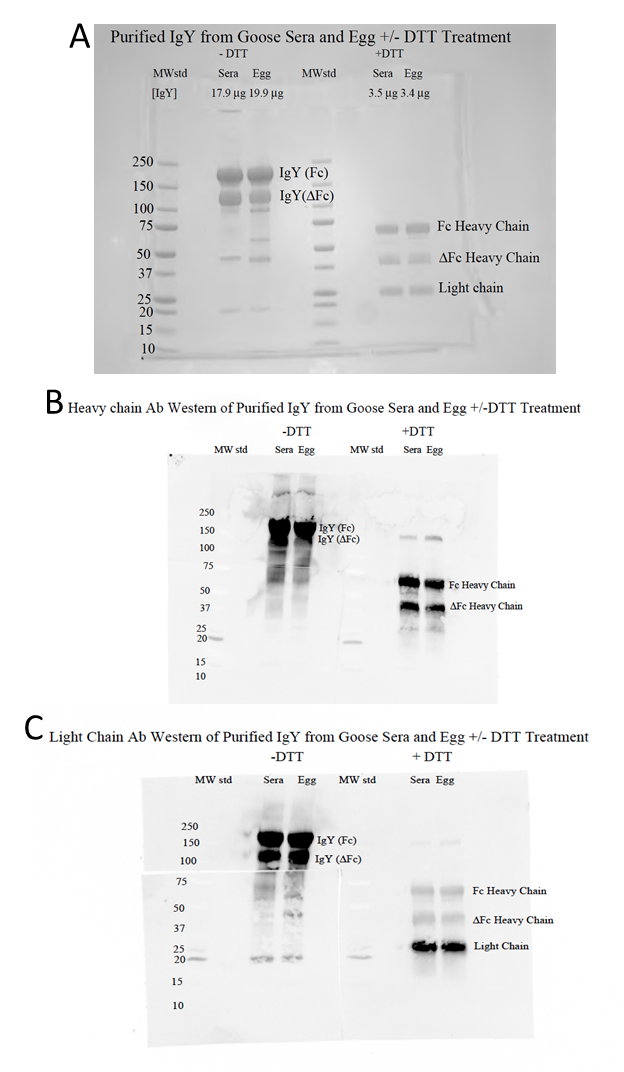

Supplement: S2 Fig — Comparison of +DTT (reduced) and–DTT (non-reduced) IgY isolated from goose sera and egg (50mM DTT) visualized by A) Coomassie stained SDS-PAGE, B) IgY heavy chain Western blot, or C) IgY light chain Western blot. Note IgY concentration listed on Coomassie stained gel in A) was used for Western blots in B) and C). SDS-PAGE gel was transferred onto Immobilon-FL (Millipore), blocked with 1% BSA in TTBS, then incubated with 1:2000 dilution of either B) rabbit α-IgY heavy chain IgG or C) rabbit α-IgY light chain IgG. This was followed by a goat α-rabbit IgG linked to a biotin and a streptavidin linked to Q-dot 625 (Invitrogen). Signals were captured using AlphaView software and AlphaImager HP Imaging System (Alpha Innotech). (TIF) [file pntd.0003803.s002.tif]

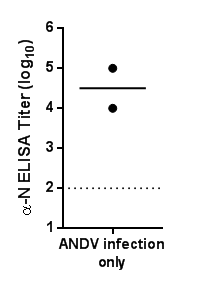

Supplement: S3 Fig — Serum from the two hamsters surviving to day 28 following ANDV challenge were analyzed by N-ELISA (see Materials and Methods). Each symbol represents and individual animal. The limit of detection, a titer of 100, is shown as a dotted line. (TIF) [file pntd.0003803.s003.tif]
